# Characteristic of Essential Oils Extracted from the Industrial-Scale Processing By-Products of Agro-foods

**DOI:** 10.1007/s13668-025-00624-5

**Published:** 2025-02-17

**Authors:** Hülya Güҫlü

**Affiliations:** Turkish Energy, Nuclear and Mineral Agency, 06510 Ankara, Turkey

**Keywords:** Agro-food waste, By-product, Essential oil, Fatty acid, Waste valorization

## Abstract

**Purpose of Review:**

Nowadays, more interest has been focused on the efficient use of agricultural and food processing post-products. The remaining wastes accumulate in large quantities, resulting in a burden on the environment. However, they possess potentially valuable compounds, making them suitable to recover essential oil from solid waste. This review evaluates the production of essential oils from apple, citrus fruits, sour cherry, corn, rice, wheat, and barley processing wastes, highlighting high levels of bioactive compounds such as tocopherols, phytosterols, and unsaturated fatty acids, which contribute to their antioxidant and antimicrobial properties.

**Recent Findings:**

Agro-food processing wastes are composed of biologically active compounds including essential oil with different functional characteristics such as antibacterial and anti-angiogenic properties. This could be due to the presence of bioactive compounds including phytosterols, tocochromanols, tocopherol, and carotenoids, as well as the natural antioxidants and the higher amount of unsaturated fatty acids.

**Summary:**

Agro-food processing by-products are a valuable source of essential oil, which is rich in mono-unsaturated and poly-unsaturated fatty acids as well as bioactive compounds. Findings provide a reference for the development of healthy products and open a new horizon for innovative and profitable utilization of the wastes generated from the industrial-scale processing of agro-foods.

## Introduction

Waste management is an important approach in developing and developed nations [1, 2]. Nowadays, an enormous amount of agro-food waste produced from industrial-scale processing of fruit and crops can lead to contamination and environmental concerns due to inappropriate disposal [2, 3]. Fruit and vegetable processing by-products such as pomace, seed, peel, etc. are cost-effective and abundant. The by-products are still rich in bioactive compounds that could be reused or recovered for food and pharmaceutical applications [4, 5]. Therefore, valorization of remaining solid wastes into value-added products can reduce the severe environmental and public health drawbacks of disposing of wastes in landfills including producing greenhouse gases (methane and carbon dioxide) by landfills used for food disposal, vulnerable to uncontrolled fermentation, and groundwater contamination [3, 6]. Agro-Food wastes are rich sources of dietary fibers, phenolic compounds, and other biologically active metabolites such as nutritional compounds, minerals, antioxidants, and essential oils with a high content of monounsaturated fatty acids (mainly omega-9 oleic acid), and PUFA (mainly omega-6 linoleic acid). Therefore, they could be a valuable candidate for recovering high-value products like essential oil. These by-products offer a unique opportunity for sustainable valorization through the recovery of essential oils, which are rich in bioactive compounds and hold potential applications in food, cosmetics, and pharmaceuticals [7, 8].

The essential oil is a complex mixture of phytochemicals such as sesquiterpenes, monoterpenes, and phenylpropanoids, which imposed a great deal of interest due to their wide spectrum of activity, low non-target toxicity as well as exhibiting suitable antioxidant, pharmaceutical, and antimicrobial activities [9, 10]. Recent studies focused on extracting essential oils from agro-food processing wastes including recovering grape seed oil, tomato seed oil, apricot kernel oil, apple seed oil, citrus peel, and seed oil, etc. [10–14].

This novel review collectively presents the current status and recent advances in the useful conversion of low-value waste and by-products generated in the food industry to upstream extraction of essential oil towards maintaining food resources as well as implementing new health-promoting food products. This study also investigates the characteristics of the essential oil recovered from apple, citrus, sour cherry, corn, wheat, rice, and barley processing waste with respect to the different extraction methods. More importantly, this paper aims to outline the concept of utilizing industrial-scale agro-food by-products for the production of edible oil with health-promoting properties, focusing on extracted oil characteristics.

## Agro-Food Processing By-Products

### Apple

In the last few years, apples have been one of the favorite fruit crops worldwide, which has received considerable interest from millions to meet health and nutritional needs [5]. Apple is commonly grown in temperate regions and is marketed as fresh fruit (71%) or processed into value-added goods (20%), including apple juice, wine, apple cider, etc. [15]. Industrial processing of the apple has led to the production of higher amounts of waste discarded in landfills, leading to groundwater contamination following serious environmental drawbacks [16]. Apple waste comprises apple seeds, seed chambers, pomace, calyx, peel, stems, and the rest of the tissue (approximately 25% of the fresh fruit’s weight) [17]. Apple pomace is a valuable source of polyphenols, dietary fiber, and minerals, while apple seeds (2–3% of the dry material) are known as a source of protein and lipid (about 29%), mostly located in the endosperm, depending on variety [18]. In this regard, apple seed oil (ASO) shows a high level of linoleic acid (around 44%) as well as large quantities of oleic acid (from 28 to 38%) and saturated acids (from 7 to 9%) [19]. ASO also exhibits peroxide and acid values of around 15 mmol O_2_/g and 4.0 mg KOH/g, respectively [8, 19]. Moreover, this essential oil represents antibacterial and anti-angiogenic properties, which could be due to the presence of bioactive compounds including phytosterols, tocochromanols, tocopherol (ranges from 50–60 mg/100 g oil), squalene, phloridzin, carotenoids, and quercetin-3-galactoside as well as the natural antioxidants and the higher amount of unsaturated fatty acids [10]. ASO is considers hypocholesterolemic and contains higher linoleic acid and oleic acid (ratio of PUFA/SFA: approximately 7), which is similar to that in grape seed oil (7.3) and rapeseed oil (7) [8]. In this regard, Bada et al. characterized the lipid profile of apple seed oil from the seeds of seven apple species from Asturias (Table 1). Among different varieties, *Collao* (22.73 ± 0.81 g/100 g of seed), followed by *Raxao* (20.19 ± 0.74 g/100 g seed), and *Riega* (19.67 ± 0.85 g/100 g of seed) seed showed higher oil content. As well, *Limón Montés* and *Riega* exhibited linoleic acid as the main component around 60.78 ± 3.07% and 60.01 ± 3.41%, respectively, as well as LLP (41.17 ± 1.98–39.32 ± 1.66%) and LLL (27.12 ± 1.32 − 17.80 ± 1.96%) as the main triglycerides. However, the highest content in total sterols belonged to the *Solarina* seed oil (558.52 ± 9.42 mg/100 g of oil), and the lowest amount was found in *Blanquina* seed oil (166.55 ± 1.89 mg/100 g of oil). *Blanquina* and *Collaos* varieties showed phosphatidylcholine around 70.58 ± 3.85% and 55.55 ± 2.96%, respectively, while high levels of β-tocopherol and α-tocopherol were evaluated in *Raxao* (125.29 ± 12.62) and *Limón Montés* (84.68 ± 5.61 mg/kg of oil), respectively [20].

**Table 1:** The different varieties, extraction method, and major findings of fruit processing by-products

Fruit processing by-products	Varieties	Extraction method	Major Findings	Reference
Apple	Blanquina, Raxao, Collaos, Durona, Riega, Solarina, and Limón Montés	Soxhlet method using a hexane as solvent	-Obtained oil contented high levels of sterols (β-sitosterol), polyunsaturated fatty acids C18:2, tocopherols (α-tocopherol), and phospholipids (phosphatidylcholine)	[20]
	Custard apple (*Annona squamosal* L.)	Solvent extraction method using methanol: chloroform (1:2)	-Good source of crude oil and crude protein -Rich in macroelements -The extracted oil was classified as a semi-dry oil	[21]
	Malus species (*M. soulardii*, M. *Bernu prieks*, M. “Ola”, M. *Berzukroga dzeltenais*) and Dessert apples of cv. “Gita”	Soxhlet extraction method using 100 mL of petroleum ether for 3 h at 70 ◦C	-Oils recovered from by-products of *Malus* spp. were found in bound forms, in the form of di- and triglycerides, and fatty acid alkyl esters -The oils recovered from by-products of *Malus* spp., especially *M. Bernu prieks*, followed by M. cv. “*Ola*”, and *M. Soulardii* are rich in Ts −11 compounds including five phytosterols and six triterpenes were identified and quantified in the tested pomace oils	[22]
	Red Delicious, Golden Delicious, Royal Delicious, and Red Chief	Soxhlet extraction method using n-hexane at 60 ◦C for 5 h	-The presence of unsaturated fatty acid in high concentration with good antioxidant potential and in vitro cytotoxic activity -The physicochemical properties of apple seed oil were comparable with edible food oil	[23]
	Acidic cider apple varieties	Soxhlet extraction method using hexane for 10 min at room temperature	-Apple seed oils were constituted of unsaturated fatty acids (90%) and high levels of tocopherols with an important antioxidant activity	[24]
	Seeds of six crab apple cultivars (inter-species hybrids of Malus sp.) (‘Kerr’, ‘Kuku’, ‘Riku’, ‘Ritika’, ‘Ruti’ and ‘Quaker Beauty’), and five dessert apple cultivars (Malus × domestica) (‘Antej’, ‘Beforest’, ‘Kent’, ‘Sinap Orlovskij’ and ‘Zarja Alatau’)	Soxhlet extraction method with n-hexane using the ultrasound technique (5 min, 35 ◦C)	-Obtained oils were rich in bio-compounds, particularly unsaturated fatty acids including linoleic acids and oleic acids (90% of the total fatty acids) as well as phytosterols (sitosterol) -A high variation in oil yield as well as contents of phytosterols and squalene, and lower fatty acids composition may be found in seed oils recovered from different apple cultivars	[25]
	Fuji and New Red Star	Soxhlet extractor was executed in a water bath for 5 h with petroleum ether	-Apple seed oil possessed interesting properties, emerging from its chemical composition -The apple seed oil was active against bacteria and mildew, and less sensitive to yeasts -The MIC of apple seed oil ranged from 0.3 to 0.6 mg/ml	[26]
	Red Fuji	Soxhlet extraction method using petroleum ether (100–110 °C for 5 h)	−11 types of fatty acids were found in apple seed oil -Oils had high levels of unsaturated fatty acids (70.598 g/ 100 g apple seed oil)	[27]
Citrus fruits	Bitter orange, lime, lemon, pomelo, grapefruit, mandarin, and sweet orange	Soxhlet extraction method	-Citrus peel essential oil showed acceptable antioxidant activity and could inhibit glucosidase and amylase	[28]
	Lemons (*Citrus limon*)	Steam distillation over a 3 h period	- Lemon peel oil could be used as a potential natural antioxidant as well as an antimicrobial compound in the food industry	[29]
	Lemon and orange	Soxhlet extraction method using hexane and ethanol	-N-hexane was a better solvent for extracting oil from lemon and orange peels than ethanol -Limonene content in lemon peel oil was lower than in orange peel oil -The highest yield of limonene (using hexane solvent) was obtained from orange peel comparing lemon peel	[30]
	Citrus limon L. (*Ku¨tdiken* variety)	Cold press method operated at 30 rpm screw rotation speed, 10-mm exit die, and maximum 40 °C oil exit temperature -Solvent extraction method using hexane (mixed at 1:2.5 (w/v) ratios, placed for 3 h at 45 °C in a water bath)	-The cold press (CP) method yielded lower oil comparing the solvent extraction (SE) method -CP oil was more turbid and less yellow than the EV counterparts -B-sitosterol, campesterol D-5-avenasterol, and stigmasterol were the most abundant sterols in both oil samples -The a-tocopherol content was significantly higher in CP sample	[31]
	Citrus japonica (kumquat)	Solvent extraction method using hexane	-Monoterpene hydrocarbons were the dominant compounds in both oils of peel and kernel -The extracted essential oil contained five monoterpene hydrocarbons which were characterized by β-phellandrene limonene, β-myrcene, α-pinene, ( +)- carvone, and six sesquiterpenoids characterized by germacrene b, germacrene D, δ-cadinene, γ-elemene, γ-cadinene, γ-muurolene and two oxygenated monoterpenes which were characterized by geranyl acetate and ( +)- carvone	[32]
	Kütdiken lemon variety (*Citrus limon* L.)	-Oil obtained using cold-press at 30 rpm screw rotation speed, 10 mm exit die, and a maximum 40 °C oil exit temperature -Solvent extraction method using hexane by mixing the ground seeds and hexane at 1:2.5 (w/v) ratios for 3 h at 45 °C in a water bath	−5 phenolic, 8 flavonoids, carotenoids, and chlorophyll were characterized in the lemon seed oils -Concentrations of naringin, syringic, and gallic acids were significantly higher in the cold-pressed oil -For both hexane-extracted and cold-pressed oils, eriocitrin, hesperidin, naringin, neohesperidin, gallic acid, and trans-ferulic acid were the most common bioactives quantified	[33]
	Lemon seeds (Citrus *limon*), orange seeds (*Citrus sinensis*), and grapefruit seeds (*Citrus paradise*)	-Oil obtained using cold-press at 30 rpm screw rotation speed, 10 mm exit die, and a maximum 40 °C oil exit temperature -Solvent extraction method using hexane by mixing the ground seeds and hexane at 1:2.5 (w/v) ratios for 3 h at 45 °C in a water bath	-All oils had some antimicrobial activities against 15 pathogenic bacteria, and some had anti- microbial activity against yeasts	[34]
	Orange varieties (Hamlin, Natal, Perario, and Valencia)	Using pressing in a vegetable oil extractor, at room temperature, with an initial rotation of 25 Hz and final rotation of 60 Hz	-Extracted oil presented high levels of α-tocopherol, carotenoids, phytosterols, and phenolic compounds as well as free radical scavenging capacity -The antiradical efficiency of the oils analyzed followed a decreasing order: Perario > Hamlin = Natal > Valencia	[35]
	Cultivars of Citrus sinensis(Valencia, salustiana, Moro, Moro Solarino, Sanguinello, Sanguinello Moscato 1, Saguinello Moscato 2, Navelina, Tarocco Tradizionale, Tarocco Dalmuso, Tarocco Gtallo, Thomson Navel)	-Steam distillation -Solvent extraction method using hexane	−54 compounds were identified, including 20 hydrocarbons (11 monoterpenes; 9 sesquiterpenes), 15 alcohols (8 monoterpenes, 4 sesquiterpenes and 3 aliphatics), 10 aldehydes (4 monoterpenes, 2 sesquiterpenes and 4 aliphatic), 4 mono-terpenic esters, 2 ketones (1 monoterpene; 1sesquiterpene), 2 oxides (1 monoterpene; 1 sesquiterpene), and one other compound	[36]
	Lemon (*Citrus limon* L.), grapefruit (*Citrus paradisi Macfayden*)	Cold press method	-Limonene, β-pinene, γ-terpinene, myrcene, sabinene, α-pinene, p-cymene, neryl acetate, and geranial were the main compounds of extracted oil −27 components were identified in the grapefruit seed oil; representing 98.6% of the whole oil	[37]
	Grapefruit (*Citrus paradisi*)	Solvent extraction method	-The extracted oil showed good antioxidant properties	[38]
	Grapefruits (*Citrus paradisi*)	Hydrodistillation method	-D-Limonene was the main compound in the obtained EO -Obtained oil showed bacteriostatic properties against the growth of *Vibrio vulnificus Salmonella parathypi* A, and *Seratia liquefaciens*	[39]
	Grapefruit (*Citrus Paradisi*. L)	-Solvent-free microwave extraction and hydrodistillation	-The extracted oil showed a wide spectrum of antimicrobial activities and 25 components -Limonene was observed as dominant for two extraction methods	[40]
	Grapefruit (*Citrus paradise* var. red blush)	Hydro-distillation method for two h at 100 °C	-Obtained oil was eco-friendly and could be a substitute for BHT, BHA sodium nitrite, and other synthetic antioxidants, nitrate, or benzoate which was commonly used in food preservation	[41]
	Grapefruit (*Citrus paradisi* L.)	-Cold press method with 30 rpm screw rotation speed, 10 mm exit die, and maximum 40 °C oil exit temperature	-The extracted oil was rich in bioactive and aromatics but has a distinct bitter and astringent flavor -The oil could be used to enrich edible oils or foods for functional purposes to provide citrus phenolic acids and flavonoids	[42]
Sour cherry	*Prunus Cerasus* L	-Solvent extraction method using n-hexane with and without ethanol (3.0%) -Supercritical carbon dioxide with and without ethanol (3.0%) at 60 ◦C and 300 bar	-The extracted oil was rich in bioactive compounds like tocopherols, polyunsaturated fatty acids, phenolic compounds, and β-carotene	[43]
	*Cerasus vulgaris Miller*	Cold press method (T: 35 °C–40 °C)	-The recovered oil presented high levels of lipophilic bioactive compounds including anthocyanins, fatty acids, tocopherols, carotenoids, and sterols -The amygdalin content of sour cherry kernel oil was not detectable	[44]
	Cultivars of sour cherry (P. cerasus L.): Koral, Wołyńska, Lucyna, Montmorency, Wróble, Wanda, Naumburger, and Wigor	Soxhlet extraction method using diethyl ether in a water bath for 6 h	-Obtained oil was a high oleic–linoleic oil with higher levels of bioactive compounds including polyphenols, tocopherols, and carotenoids	[45]
	*Cerasus vulgaris Miller*	Cold press method (T: 35 °C)	-The growth of *Escherichia coli, Staphylococcus aureus*, *Salmonella typhimurium*, and *Listeria monocytogenes* were inhibited	[46]
	*Prunus cerasus* L	Cold-press extraction method (T: 50 °C)	-The obtained oil showed low-level of peroxide value and high levels of TPC and AC -The obtained oil samples were rich in linoleic, oleic, and linolenic acids	[47]
	Prunus *cerasus* L	Cold-press extraction method	-The extracted oil was composed of oleic and linoleic acids, α-eleostearic acid, octadecatrienoic, and palmitic acid	[48]
	Prunus cerasus L	Soxhlet method using petroleum ether for 4 h	-The fatty acids profile showed a high content of linoleic acid and oleic acid	[49]
	Six cultivars of (*Prunus cerasus*L.):‘Bulatnikovskaya’,‘Haritonovskaya’, ‘Latvijas Zemais’, ‘Shokoladnica’, ‘Tamaris’ and ‘Zentenes’	Solvent extraction method using n-hexane follows by treating with ultrasound in the ultrasonic bath	-The recovered oils were rich in lipophilic bioactive compounds including sterols, essential fatty acids, squalene, tocopherols, and carotenoids	[50]
	Sweet cherry (*P. avium* L*.*) and sour cherry (*P. cerasus* L.)	Cold press method (20 rpm screw rotation speed, 14 mm exit die, and 45 °C oil exit temperature)	-Showed high levels of unsaturated fatty acids and bioactive compounds	[51]

Eshra et al. examined the oil extracted from the Custard apple seed kernel (*Annona squamosal* L.) and demonstrated that the oleic, linoleic, palmitic, and stearic acids (at the range of 49.75%, 22.50%, 15.06%, and 4.63%, respectively) were the dominant fatty acids of custard apple seed kernel oil. As reported, the oil could be characterized as a semi-dry oil in which triacylglycerols were the major class of lipid fractions [21].

Radenkovs et al. studied the oils recovered from the waste of *Malus* spp, which was rich in unsaturated fatty acids including linolenic (57.8%), α-linolenic (54.3%), and oleic (25.5%) acids. As reported, the *Malus* species and dessert apple oils represented considerable content of total tocopherols (16.8 to 30.9 mg/ mL), while *M. Bernu prieks* showed a significantly higher amount of δ-tocopherol. Based on the results, pomace oil was composed of β-Sitosterol (a percentage distribution of 10.3 to 94.5%) and lupeol (0.1 to 66.3%) as the main triterpene [22].

Walia et al. examined apple seed oil and found oleic (46.50%) and linoleic acid (43.81%) as the main fatty acid composition. In this regard, apple seed oil revealed high saponification (184.91 mg KOH/ g oil) and iodine value (121.8 g I/ 100 g). The obtained oil represented antioxidant activity (40.06 µg/ mL) as well as the refractive index, acid value, and relative density around 1.47 mg/ mL, 4.28 mg KOH/ g, and 0.97 mg/ mL, respectively. The antioxidant potential (IC50) of apple seed oil was evaluated as 40.06 µg/mL [23].

Rodríguez Madrera and Suárez Valles derived oil from the by-product (Apple seeds) of the cider industry. The oil extraction yielded 19.7%, which contained 90.3% unsaturated fatty acids (linoleic acid (55.3 ± 1.2%), linolenic acid (1.2 ± 0.1%), oleic acid (33.4 ± 0.8%)) as the major fatty acids. ASO also included high levels of tocopherols (1280 ± 104.8 mg/kg oil), α-tocopherol (439.2 ± 34.5 mg/kg oil), and β-tocopherol (794.5 ± 62.2 mg/kg oil) as well as DPPH radical scavenging activity [24].

Górnaś et al. studied the profile of lipophilic compounds of six crab and five dessert apple cultivars seed oils. The apple seeds oil yielded ranged around 12.06 to 27.49 g/100 g dry weight base, which was higher by 30% from crab apple seeds comparing dessert apple seeds. The extracted oil composed of linoleic acid (59.37–67.94%), oleic acid (20.68–29.00%), palmitic acid (5.78–8.33%) as well as phytosterols (1.13–7.80 mg/g), β-sitosterol (51–94% of total phytosterols) and squalene (0.01–0.34 mg/g) [25].

Tian et al. utilized *Fuji* and *New Red Star* species apple seeds to recover their essential oils. The extracted oils exhibited a density (25 °C) of 0.902–0.903 mg/ml, an acid value of 4.036–4.323 mg KOH/g oil, an iodine value of 94.14–101.15 g I/100 g oil; refractive index of 1.465–1.466; and saponification value of 179.01–197.25 mg KOH/g oil as well as linoleic acid (50.7–51.4 g/100 g), oleic acid (37.49–38.55 g/100 g), palmitic acid (6.51–6.60 g/100 g), arachidic acid (1.49–1.54 g/100 g), and stearic acid (1.75–1.96 g/100 g) [26].

Yukui et al. could successfully recover 291 g/kg oil from apple seeds (cv. *red Fuji*) and reported c18:1, c18:0, and c16:0, approximately 43.03 g/100 g oil, 26.47 g/100 g oil, and 5.60 g/100 g oil, respectively as the main fatty acids. As reported, c16:1, c18:3, c20:1, c20:2, c22:0, and c24:0 were detected in apple seeds oil, revealing a large quantity of unsaturated fatty acids (70.598 g/100 g) in apple seeds oil [27].

### Citrus Fruits

Citrus fruits belong to the *Rutaceae* family, produce about 100 million tons worldwide annually, and are known as a valuable source of antioxidants such as flavonoids, ascorbic acid, and phenolic compounds as well as essential oils [52, 53]. Citrus fruit industrial processing led to remain 50–60% of the fruit mass as peels, membrane, and seed residue, which is either incinerated, composted, landfilled, used as animal feed and pectin production, or typically discarded and represents an environmental challenge for the waste industry [11]. Although citrus peel waste possesses high amounts of essential oil, it contains a low pH (3.4) and nitrogen content, which prevents fast decomposition as well as increasing groundwater pollution due to antimicrobial properties and percolation that lead to the detriment of soil microorganisms [3]. Citrus peel oil (CPO) extraction could be the best way to reduce waste generation while minimizing environmental drawbacks [53]. CPO is commonly used as preservatives and flavoring agents in the food industry due to containing a natural and antioxidant, anti-glucosidase, and cost-effective substitutes including linalool (18.26%–29.08%), linalool acetate (17.17%–30.47%), limonene (17.08%–22.44%), α-terpineol (6.08%–11.06%), β-ocimene (1.52%–5.02%), geraniol (2.05%–6.30%), nerolidol (2.93%–4.00%), farnesol (2.08%–2.97%), geranyl acetate (1.89%–2.80%), and β-pinene (2.71%–3.29%) [54, 55]. Accordingly, Heydari Koochi et al. revealed that CPO showed glucosidase (475–780 mg acarbose equivalent/g), antioxidant capacity (375–643 mg Trolox equivalent/g) as well as strongly inhibited amylase (520–738 mg acarbose equivalent/g) properties [28]. Imran et al. used a solvent extraction method to extract grapefruit peel oil (GPO). The GPO was characterized by refractive index (1.47), specific gravity (0.85), pH (3.67), acid value (0.81), and iodine values (99.4) [38].

Moosavy et al. used lemon peel essential oil (LPO) as an antimicrobial and antioxidant compound in the food industry. Based on the results, LPO showed 55.09% inhibition of DPPH. Additionally, the MIC and MBC values of LPO against *S. aureus* were 1.25 and 5%, respectively. LPO also showed the total amount of flavonoids and phenol content around 11.72 ± 1.82 mg/g rutin equivalent, and 81.82 ± 8.02 mg gallic acid equivalent/g of the LPO, respectively [29].

Karne et al. investigated orange peel essential oil (OPO) and lemon peel essential oil (LPO) extracted from peel zest and dried peel samples. As reported, higher oil extraction was obtained from orange peel zest samples than dried orange peel samples due to the vaporization of limonene through heating the peels. Moreover, lemon peels showed lower limonene comparing orange peels. The study concluded that peels contain 28 volatile substances such as alcohol (50%), terpene compounds (32%), and formaldehyde (18%), as well as β-pinene and γ-terpinene (around 5–12% and 5–10%, respectively) [30].

Yilmaz and Aydeniz Güneşer determined the physicochemical and thermal characteristics of lemon seed oils (LSO), which were recovered using cold-press and hexane-extraction methods. Based on the results, hexane extraction (71.29%) yielded higher oil, peroxides, p-anisidine, and free fatty acids comparing the cold press method (oil yield of 36.84%). The cold-pressed oil showed linoleic acid, palmitic acid (as the major fatty acids), campesterol, and β-sitosterol (as the dominant sterols), as well as higher α-tocopherol, total phenolics, and antioxidant capacity. As concluded, the cold press method led to obtaining high-quality LSO, which could be used in the chemical, food, and cosmetic industries [31].

Nouri and Shafaghatlonbar evaluated the constituents of *Citrus japonica’s* essential oils (CJO) by GC and GC/MS. Findings showed that the content of essential oil in kernel and peel was 0.8% and 1.1 based on dry weight, respectively. Essential oil derived from the peel and kernel of *Citrus japonica* revealed a higher amount of germacrene D (12.1 and 6.3%, respectively) and limonene (51.0 and 47.1%, respectively). However, the hexane extracts of its peel and kernel were characterized by a higher amount of linolenic acid (13.1 and 16.3%, respectively) and dodecanol-1(12.9 and 20.8%, respectively). Results demonstrated that both oils from different parts of *C. japonica* possessed considerable antioxidant activity [32].

Guneser and Yilmaz quantified rutin, catechin, naringenin, naringin, eriocitrin, neohesperidin, and kaempherol as flavonoids, hesperidin, and gallic, tr-ferulic, rosmaniric syringic, and tr-2-hydrocinnamic acids as phenolic acids in the lemon waste oils. Lemon seed oil was very aromatic and was characterized by citrus, herbal, terpenic, woody, and floral aroma descriptors. Cold-pressed lemon seed oil showed a significantly higher content of naringin, gallic, and syringic acids followed by higher concentrations of different aromatic volatiles. Although lemon seed oil was found unsuitable for direct edible use, its bioactive components and unique aroma made it suitable for pharmaceutical, functional food, and cosmetic applications [33].

Güneşer et al. extracted lemon (LSO), grapefruit (GSO), and orange seed oils (OSO) using the cold press method and determined their antimicrobial activity. Based on the results, the citrus oils showed inhibition zones against fifteen tested pathogenic bacteria, ranging from 6.62 to 11.00 mm. Although only LSO and OSO represented some inhibition against *Candida utilis* yeast, no measurable inhibition zone was found against *Micrococcus luteus*, using all kinds of oils. As seen, LSO obtained through cold-press and solvent-extract methods (at 2% level) could significantly inhibit the growth of *Staphylococcus aureus*. In comparison, OSO obtained using cold-press (16%), and microwave-treated cold press (50%) methods could successfully inhibit the growth of *Klebsiella pneumonia* [34].

Jorge et al. evaluated total carotenoids, phenolic compounds, phytosterols, tocopherols, and antioxidant activity of different orange varieties of seeds oil (OSO) including *Perario*, *Hamlin*, *Valencia*, and *Natal*. Based on the results, the lowest and highest antioxidant activity among the OSO belonged to the *Natal* (56.0%) and *Perario* (70.2%), respectively. As well, the extracted oils showed valuable content of total phenolic compounds, phytosterols, total carotenoids, and α-tocopherol in the range of 4.43 g/kg, 1304.2 mg/kg, 19.01 mg/kg, and 135.65 mg/kg, respectively [35].

Geraci et al. investigated the essential oils extracted using steam distillation and hexane methods from the peel of 12 cultivars of the orange from central-eastern Sicily. Based on the results, the main component in all samples was d-limonene (73.9–97%) and a low amount of nerol, geraniol, and linalool. As found, ‘*Solarino Mor*o’ and *'Sanguinello’* essential oils were remarkably active against *L. monocytogenes*. However, ‘*Valencia’* essential oil extracted using the hexane method showed antimicrobial activity against all the tested micro-organisms including *Listeria monocytogens*, *Staphylococcus aureus*, and *Pseudomonas aeruginosa* [36].

Kirbaşlar et al. studied lemon and grapefruit peel oils extracted using the cold-press method. Although lemon peel oil (LPO) showed 42 different compounds, grapefruit peel oil (GPO) represented 27 components. In this regard, LPO showed a high content of monoterpene hydrocarbons (89.9%) as the first major component as well as limonene, γ-terpinene, and β-pinene around 61.8%, 10.6%, and 8.1%, respectively. Similarly, monoterpene hydrocarbons (96.4%) were found to be the major component in GPO followed by limonene (92.5%) and myrcene (2.6%). However, GPO contained a lower amount of aldehydes, alcohols, sesquiterpene hydrocarbons, and esters than that in LPPO [37].

Özogul et al. used GC–MS to evaluate the characteristics of GPO, which showed d-limonene (82.86%) as the major compound. The GPO showed bacteriostatic properties against the majority of tested bacterial strains (*Klebsiella pneumonia*, *Staphylococcus aureus*, *Salmonella Paratyphi A*, and *Enterococcus faecalis*); however, increasing the GPO up to 25 mg/mL revealed the inhabitation effects on the growth of *Salmonella parathypi A*, *Seratialique faciens*, and *Vibrio vulnificus* [39].

Uysal et al. obtained the grapefruit peel essential oil (GPO) using hydrodistillation (HD) and solvent-free microwave extraction (SFME) methods. Results identified twenty-five components in the GPO which detected limonene (91.5–88.6%) as dominant and linalool (1.1–0.7%), β-pinene (0.8–1.2%), and α-terpinene (0.7–1.0%) as the other minor components for two extraction methods, respectively. Results of the disc diffusion method showed that GPO had a wide spectrum of antimicrobial activities against *Staphylococcus epidermidis*, *Salmonella typhimurium*, *Staphylococcus aureus*, *Escherichia coli*, *Enterococcus faecalis*, *Serratia marcescens*, and *Proteus vulgaris*, with their inhibition zones ranged from 11 to 53 mm [40].

Ghani et al. indicated that the oil content and its composition significantly changed during different fruit maturation stages. In this regard, the most abundant components in all stages of grapefruit EO are known as monoterpene hydrocarbons (93.87–96.80%) as well as limonene (90.92–93.98%) [41].

Yilmaz et al. produced grapefruit seed oil (GSO) using the cold press method. It was shown that GSO contains β-sitosterol (80%), linoleic acid (40%), and α-tocopherol (220–320 mg/kg oil) as the major components as well as the rutin, naringin, neohesperidin, hesperidin, tr-ferulic and gallic acids as dominant flavonoids and phenolic acids [42].

### Sour Cherry

Sour cherry (*Prunus Cerasus* L.) is a popular fruit across Asia, Europe, and North America, and belongs to the *Rosacea* family and *Prunoideae* subfamily [46]. Sour cherry is commonly consumed as fresh fruit, frozen pitless sour cherry, processed products including sour cherry juice, syrup, jam, canned and dried products due to its valuable source of nutraceuticals and phytochemicals, including anthocyanins with bioactive properties [43]. However, the large amounts of kernels known as pit or stone were produced as waste during the industrial-scale processing of sour cherries (about 7–15% of the total fruit weight) with limited utility, which is used as animal feed or is discarded, creating an important disposal problem for the food industry. The sour cherry kernel is a valuable source of protein, dietary fiber, fat (17–36%), antioxidants, and phenolic compounds [56]. Moreover, the sour cherry kernel contains B vitamins group (B_1_, B_3_, B_5_, and B_6_), minerals such as potassium, calcium, and amino acids (such as glutamic acid and lysine) [57]. Sour cherry kernels contain about 32–36% oil, which is rich in mono-unsaturated and poly-unsaturated fatty acids with high content of oleic acid (50–53%) and linoleic acid (35–38%), β-tocopherol, γ-sitosterol, as well as bioactive and valuable compounds [58, 59]. Based on the literature, SCKO shows health-promoting benefits for humans due to its antimicrobial, antioxidant, and anti-inflammatory attributes, reducing the risk of neurodegenerative diseases, cardiovascular diseases, oxidative stress, diabetes, and cancer [42].

Yılmaz & Gökmen investigated the chemical composition of the sour cherry kernel oil (SCKO). Based on the results, sour cherry kernels contained proteins (29.3%), oil (17.0%), and dietary fibers (30.3%). SCKO was extracted from the kernel using supercritical carbon dioxide (SC-CO_2_) and conventional hexane, which showed no significant effect of the extraction method on the fatty acid composition of the oil. However, the oil extracted by SC-CO_2_ contained significantly lower levels of β-carotene and tocopherols comparing oils extracted using hexane. Findings revealed an increasing trend in antioxidant capacity, β-carotene, and total phenolic content of oils obtained from both methods. Additionally, reduction in total tocopherols (9.8%) as well as an enhancement in hydroxymethylfurfural (1.4 mg/L) and total phenolic content (4.5 times) of SCKO was observed through roasting kernels at 160 ˚C for 30 min, which was comprised of oleic acid (46.3%), linoleic acid (41.5%), palmitic acid (6.4%), linolenic acid (4.6%), and stearic acid (1.2%) [43].

Yilmaz et al. obtained SCKO using the cold press method. Based on the results, SCKO obtained from kernel oil from *Prunus* species, including sweet cherry (*P. avium* L.) and sour cherry (*P. cerasus* L.), showed high levels of bioactive compounds and unsaturated fatty acids. As reported, amygdaline as a cyanogenic glycoside was found in the kernels of sour cherry (3.89 mg/g kernel) with a lethal dose of 0.5–3.5 mg/g body weight. A meager amount of amygdaline was reported in the sour cherry kernel oil obtained using the cold press method, which could be reduced by different procedures, including crushing, soaking, and drying the sour cherry kernel. It was concluded that these cold-pressed oils were not properly for direct consumption and could be used in cosmetics, nutraceuticals, and energy generation purposes [51].

Kazempour-Samak et al. used the cold press method to extract oil (4%) from the sour cherry kernel (*Cerasus vulgaris Miller*), which showed a free fatty acid value of 1.36 mg KOH/g oil and acceptable organoleptic properties. Although SCKO showed a peroxide value of 0.99 meqO_2_/kg oil and anisidine index of 0.15. The predominant fatty acids of SCKO were evaluated as linoleic acid (42.34%), oleic acid (35.45%), α-eleostearic acid (9.34%), and palmitic acid (6.54%), respectively. Furthermore, major triacylglycerols of SCKO consisted of OLL (20.44%), OOL (16.99%), LLL (8.20%), LLEl (7.28%), PLO (7.24%), OElO (5.03%), OOO (4.70%), ElLO (4.54%), PLL (4.35%), and POO (3%), respectively. Based on the results, β-sitosterol, Δ5-avenasterol, sitostanol, campesterol, and stigmasterol were obtained as 83.55%, 6.8%, 4.8%, 3.5%, and 0.53%, respectively. Also, SCKO showed higher content of anthocyanin (177.84 mg/L,), antioxidant activity (73.22%), flavonoid (46.37 mg/g dry matter), phenolic compounds (33.44 mg GA/g dry matter), tannin (1.21 mg GA/g dry matter), and tocopherol (832.5 mg/kg oil). However, no detectable amount of amygdalin was observed in the oil sample extracted from the sour cherry kernel [44].

Górnaś et al. studied the recovered lipophilic bioactive compounds of SCKO from fruit pits of six sour cherry cultivars. Based on the results, oil yielded ranged around 17.5–37.1%, with the main fatty acids (94–96% of total detected fatty acids) of linoleic (35.50–46.06%), oleic (25.25–45.30%), α-eleostearic (7.43–15.76%) and palmitic acids (5.06–7.38%). SCKO contained remarkable amount of tocochromanols (around 118.2–163.6 mg/100 g of oil), carotenoids (around 0.51–1.75 mg/100 g of oil) squalene (around 65.8–102.8 mg/100 g of oil), sterols (around 313.6–1041.3 mg/100 g of oil), and β-sitosterol (around 77–82% of the total sterol) [50].

Stryjecka et al. investigated the main components, including crude fat, protein, and oil content of eight cultivars of sour cherry (*Koral*, *Wołyńska*, *Lucyna*, *Montmorency*, *Wróble*, *Wanda*, *Naumburger*, and *Wigor*) grown in Poland. Based on the results, SCKO showed an acid value of 1.20 mg KOH/g, an iodine value of 120 g I_2_/100 g of oil, a peroxide value of 1.49 mEq O_2_/kg, and a saponification value of 184 mg of KOH/g as well as linoleic (39.1–46.2%) and oleic (25.4–41.0%), α-eleostearic (8.00–15.62%), palmitic (5.45–7.41%), and stearic acids (2.49–3.17%), sterols (336–973 mg/100 g) and squalene (66–102 mg/100 g of oil), total tocochromanols (119–164 mg/100 g oil), polyphenols (19.6–29.5 mg/100 g oil), and carotenoids (0.56–1.61 mg/100 g oil). As concluded, ‘*Naumburger’* cultivar provided the highest amounts of oil, total polyphenol, PUFA (included linoleic acid), plant sterols, α-and β-tocopherol, and total carotenoids [45].

Kazempour-Samak et al. investigated the chemical composition and antibacterial properties of SCKO (against *Staphylococcus aureus*, *Listeria monocytogenes*, *Escherichia coli*, and *Salmonella typhimurium*), extracted using the cold press method. The results showed that SCKO was rich in anthocyanin, polyphenols, tocopherol, and flavonoids. Moreover, *Listeria monocytogenes* was detected as the most sensitive among all tested microbial strains against SCKO [46].

Atik et al. used the cold press method to extract oil from sour cherry kernels. Based on the results, peroxide, free fatty acid, total phenolic content, and anthocyanin content of SCKO were evaluated as 1.70 meq O_2_/kg and 6.60%, 33.65 GAE/g, and 1.86 mmol TE/g of extract, respectively. Major fatty acids of SCKO were evaluated as oleic (72.72%) and linoleic acids (42.42%). SCKO also showed β-sitosterols (6018.27 ppm), benzoic acid (79.7 ppm), and tocopherol (224.43 ppm), indicating its potential to be used as a source of edible oil [47].

Korlesky et al. investigated the characteristics of Montmorency SCKO by various methods (NMR, GC, thermogravimetric analysis, LC–MS, differential scanning calorimetry, and X-ray powder diffraction). As reported, SCKO showed an acid value, saponification value, and unsaponifiable matter content of about 1.45 mg KOH/g, 193 mg KOH/g, and 0.72%, respectively. SCKO was comprised of oleic (O) and linoleic (l) acids as well as α-eleostearic acid and palmitic (P) acid. Additionally, SCKO showed six major triacylglycerols as OOO (16.83%), OLO (16.64%), LLO (13.20%), OLP (7.25%), OOP (6.49%), and LElL (6.16%), respectively with at least one saturated fatty acid. Moreover, SCKO showed the thermal decomposition temperature at 352 °C and the oxidative induction time of 30.3 min at 130 °C [48].

Popa et al. used an organic solvent extraction method for obtaining oil from the sour cherries from Banat. Based on the results, SCKO showed high levels of oleic (42.9%), linoleic (38.2%) acids as well as palmitic (11%) and stearic (6.4%) acids [49].

### Corn

Corn (*Zea mays* L.) is composed of the seed coat, cotyledon, and germ, which is broken down into main starch products and by-products as a low economic value animal feed [60]. Corn-husk is one of the major agricultural wastes consists of lignin (4.46%), cellulose (57.74%), and hemicellulose (34.03%) as well as about 5–12% oil which is consists of more than 80% unsaturated fatty acids [61]. These functional bioactive compounds play an important role in the improvement of human immunity, metabolism, human blood lipids, and the development of human brain function [62]. Corn husk oil (CHO) also contains phytosterol, a natural plant active component, which widely exists in various vegetable oils, seeds, and nuts with a similar structure to cyclic alcohol and is known as a precursor of vitamin D and various hormones. CHO is a rich source of sterols such as phytosterols with the potential of affecting chronic diseases inversely, anti-aging and anti-oxidation effects in the human body as well as maintaining cholesterol balance [63].

The extraction of the CHO is difficult due to the tight structure of the husk. However, the organic solvent extraction and pressing methods were successfully used as the traditional preparation methods for extracting CHO. In this regard, the subcritical extraction solvent method was used in some studies which were pollution-free, harmless, non-toxic, cost-effective, and showed no distractive effects on bioactive compounds existed in CHO [63]. In this regard, Taylor and King investigated the development of systems using supercritical carbon dioxide (SC-CO_2_) at various temperatures (40, 60, and 80 °C) and pressures (13.8, 34.5, and 69 MPa) combined with supercritical fluid chromatography (SFC) for fractionation and enrichment of high-value ferulate phytosterol esters (FPE) contained in corn bran oil (Table 2). As reported, extraction conditions: 34.5 MPa at 40 °C and 69 MPa at 80 °C showed the greatest percentage of FPE (∽1.25% FPE) [64]. As well, Ramjiganesh et al. reported a similar fatty acid profile of CHO compared to corn oil; however, CHO contained ferulate esters (6.7%), phytosterols (13.9%), free sterols (1.3%), and steryl esters (5.9%) [65].

**Table 2:** The extraction method and major findings of cereal processing by-products

Cereal processing by-products	Extraction method	Major Findings	Reference
Corn	Supercritical fluid extraction method using supercritical carbon dioxide (SC-CO_2_) at various pressures (13.8, 34.5, and 69 MPa) and temperatures (40, 60, and 80 °C)	-Extraction conditions: 34.5 MPa at 40 °C and 69 MPa at 80 °C showed the greatest percentage of ferulate phytosterol esters	[64]
Rice (Oryza sativa L.)	Solvent extraction method using n-hexane at 62–63 °C.for 7 h	-Both n-hexane and isopropanol extracted oil from the rice bran -N-hexane yielded more oil comparing isopropanol	[66]
	Solvent extraction method	-The fatty acid profile analyses showed higher partial glycerides, palmitic, oleic, and linoleic acid contents, comparing the commonly used vegetables	[67]
	Cold pressing (T:65 oC), Soxhlet extraction using hexane, supercritical CO_2_ extraction, and microwave extraction	-RBO was a source of SFA, MUFA, and PUFA as well as γ-oryzanol, tocotrienols, and tocopherols	[68]
	Continuous countercurrent SC-CO_2_ fractionation	-A high temperature (80 °C) and a low pressure (138 Bar) could effectively remove free fatty acids from rice bran oil	[69]
Wheat (*Triticum aestivum* L.)	The Soxhlet extraction method uses hexane at room temperature for ∼10 h, and desolventalization at 45 °C	-The obtained oil was composed of a significant concentration of antioxidants -The oil contained polar fractions that had a good hypolipidemic effect	[70]
	Supercritical carbon dioxide at different extraction pressures (25, 40 and 55 MPa) and temperatures (40, 70, and 95 ºC)	-The highest values of extraction temperature and pressure (55 MPa and 95 ºC) provided the bran oil with the highest antioxidant capacity	[71]
	Supercritical fluid extraction in a semi-pilot plant at 25.0 ± 0.1 MPa, 40 ± 2 °C, and 8 ± 1 kg CO2/h	-The highest antioxidant capacity, alkylresorcinols, and phenolic content of WBO were evaluated at samples obtained using the SFE method at 55 MPa and 95 ºC	[72]
	Supercritical carbon dioxide at 25.0 ± 0.1 MPa and 40 ± 2 °C	- Extraction using supercritical CO_2_ has led to obtaining oil with a high content of valuable bioactive compounds as well as desirable stability during storage -This oil could be an interesting food ingredient or a natural antioxidant	[73]
	Supercritical carbon dioxide (SC-CO2) (pressure: 10 to 30 MPa; temperature: 313.15 to 333.15 K; a CO_2_ flow rate of 26.81 g/min for 2 h) and soxhlet extraction using hexane	-The oil extracted using the SC-CO_2_ method showed higher radical scavenging activity as well as lower peroxide and acid values compared to oil extracted using hexane	[74]
barley (Hordeum vulgare L.)	Solvent extraction method using n-hexane at 40℃ for 4 h	-Extracted oil showed high peroxide value and acid value, indicating that the oil should be further refined before use -The oil was a rich source of biological activity compounds, especially β-sitosterol and linoleic acid	[75]
	Solvent extraction method using petroleum (60–90 °C)	- Linoleic acid followed by palmitic acid was the main fatty acid in the oil -The results showed that the oil from the hulless barley bran could be a rich source of valuable essential fatty acids	[76]

### Rice

Rice (*Oryza sativa* L.) is one of the oldest crops that are consumed as a staple food all over the world. Rice kernel consists of endosperm (70% of the total seed weight), husk (20–21% of the total seed weight), bran (6–8% of the total seed weight), and germ (1% of the total seed weight) [77, 78]. Rice bran as a brownish outer layer of rice grains is the residue part of rice produced during the de-husking and milling process [79]. Rice bran typically contains rice germ and broken rice which constitutes about 8% of the total rice grain’s weight [80]. Generally, rice bran contains carbohydrates (around 34 to 62%), protein (11 to 15%), fat (15 to 20%) with a unique fatty acid composition, ash (8 to 12%), and dietary fiber (7 to 11%) as well as various bioactive compounds including phenolic acids (Υ-oryzanol, ferulic acid), α-lipoic acid, vitamin E (tocopherol and tocotrienol), flavonoids, squalene, octacosanol, polycosanol, phytic acid, inositol hexaphosphate and ceramides [81, 82]. Rice bran oil (RBO) contains a remarkable concentration of bioactive compounds like abundant phytosterols as well as a well-balanced composition of fatty acids (a range of 47% monounsaturated, 33% polyunsaturated, and 20% saturated), very high burning point, delicate flavor, and neutral taste which makes it a highly healthy and nutritious vegetable oil to reach the high-value utilization of rice bran byproducts [78, 81].

RBO shows various health-promoting effects including anti-obesity, anticancer, cholesterol-lowering, anti-inflammatory, antibacterial, anti-allergic, skin-protective, anti-diabetic, gastroprotective, neuroprotective, and immune-enhancing effects [83]. However, RBO is sustainable to the hydrolysis reactions as the result of the dispersion of lipases from the endosperm into the rice bran due to the disruption in the cellular structure of the rice bran layer during the processing of brown rice [84]. This led to the production of a pungent odor as the result of the rapid hydrolytic rancidity of rice bran [82].

Rice bran oil can be categorized into three types: full-fat (retains its natural oil content which has not undergone any oil extraction process), low-fat rice (contains around 7% of oil), and defatted rice bran (contains less than 1% of oil as the residue left after extracting rice bran oil using organic solvents) [83]. Rice bran can be further classified into polished (lower lipid content and rich in protein) and rough (higher lipid content with fewer milling processes) rice bran based on different techniques and the number of times of rice milling processes [85]. RBO contains saturated fatty acids (22%), monounsaturated fatty acids (41%), and polyunsaturated fatty acids (37%), while the main fatty acids are palmitic, oleic, and linoleic acids (proportions: 1:1.5:1), respectively [86]. Additionally, RBO contains high levels of tocopherols (15,300 mg/kg), phytosterols (10,500 mg/kg), and tocotrienols (205.7 mg/kg), making it suitable for utilization in different food, functional blended oils, frying oils, health supplement, and pharmaceutical industries [78]. Accordingly, Okoro et al. studied the mineral content of rice husk oil comparing commercialized vegetable oil using atomic absorption spectroscopy. According to the results, rice husk oil contained a higher concentration of magnesium, calcium, and phosphorus compared to vegetable oils which made it better for consumption [66].

Gopala Krishna et al. studied the compositions of refined RBO and showed higher values of Lovibond units for color (7.6–15.5), unsaponifiable matter (2.5–3.2%), tocopherols (48–70 mg%), free fatty acid (0.14–0.55%) as well as oryzanol (0.14 to 1.39%). Moreover, higher free fatty acid values were detected in high oryzanol RBO. The fatty acid profile analyses showed TG levels around 79.9–92% and higher partial glycerides, as well as higher palmitic, oleic, and linoleic acid contents, comparing the commonly used vegetable oils with TG levels around > 95% [67].

Ghosh et al. indicated the delicate flavor and high smoke point of RBO, which made it excellent for utilization in salad and cooking oil. As reported, RBO was rich in unsaponifiable fractions, which contained micronutrients like phytosterols, vitamin E complexes, polyphenols, gamma oryzanol, and squalene. However, the high content of acetone-insoluble and FFA in RBO made it difficult to process. In order to obtain good quality oil with low refining loss, most of the research interest has been growing in RBO processing methods [87]. Based on the literature, non-conventional methods of RBO extraction such as subcritical H_2_O, super/sub-critical CO_2_, microwave-assisted, enzyme-assisted aqueous, and ultrasound-assisted aqueous extraction methods were more efficient and eco-friendly than conventional extraction methods with potential of improving the yields as well as the nutritional profile of RBO [68].

Dunford et al. investigated the deacidification of rice bran oil using continuous countercurrent SC-CO_2_ fractionation. Based on the results, a high temperature (80 °C) and a low pressure (138 Bar) could effectively remove free fatty acids from rice bran oil with no loss in oryzanol content in the extract [69]. Yu et al. (2019) showed that the phytosterol conversion reached 93.2%, while the free fatty acid content of RBO reduced (1.1%) when the CO_2_ filling pressure of the supercritical CO_2_ method was 8.0 MPa [88]. In addition, enzymatic deacidification of RBO through the tandem continuous-flow reactors showed a 40% higher retention rate of γ-oryzanol comparing the traditional alkaline refining method [89].

### Wheat

Wheat (*Triticum aestivum*) is a major cultivated cereal around the world which leads to the production of high amounts of wheat bran (WB) as the by-product of wheat milling processing [90]. WB is the outer layer of the wheat kernel and has food and nonfood applications [91]. Evidence indicates that WB is a valuable source of biologically active compounds including nonstarch carbohydrates (55–60%), minerals (3–8%), protein (13–18%), starch (14–25%), B vitamins, alkylresorcinols (biological activities such as antioxidant, antibacterial, enzyme inhibitor, antifungal, activities, and cancer prevention), steryl ferulates (biological activities such as anti-inflammatory, hypocholesterolemic–hypolipidaemic and antioxidants activities), other polyphenols and fat (3–4%), providing health-promoting properties to consumers [70, 92]. As documented, WB oil possessed bioactive compounds and was composed of unsaturated fatty acids (oleic and linoleic acids) with inhibitory characteristics against colon carcinogenesis. Therefore, fortification of defatted bran diet with WB oil could remarkably inhibit colon tumorigenesis, whereas omission of lipids and lipid-soluble compounds (sterols, oryzanol-like compounds, tocopherols, and carotenoids) from WB, increased it. In this regard, Talawar et al. investigated the nutrition and quality properties of dark compound chocolate containing WBO concentrate (0.5, 1, 1.5, and 2%). Based on the results, the addition of the WBO concentrate in the formulation of the dark chocolate led to the softened texture, increased in free fatty acids (FFA) (3.56, 3.61, 3.65, and 3.68), and a degree of unsaturation (2.42, 3.10, 3.41, and 3.8), with no changes in color compared to the control chocolate, providing the potential of WBO to be used as the nutraceutical supplements in food products [93].

Talawar et al. studied two methods to obtain WBO concentrates, using silicic acid coupled with acetone (WBA) and hexane (WBH). As a result, WBA was enriched with fat-soluble bioactives (sterols, tocopherols, oryzanol-like compounds, and carotenoids) that exhibited enhanced color and viscosity. Otherwise, WBA showed limited DNA and LDL oxidation, higher free radical scavenging activity, and inhabitation of HMG-CoA reductase and lipase activity better than WBH [70].

Rebolleda et al. successfully obtained WBO using a supercritical fluid extraction method due to several advantages such as the possibility of using CO_2_ as the GRAS and cheap solvents, short extraction times, high selectivity, which gives rise to extracts containing phenolic compounds, alkylresorcinols, and tocopherols with a desirable tyrosinase inhibitory and antioxidant activities [72].

Rebolleda et al. investigated the potential of supercritical fluid extraction (SEF) at different extraction pressures (25, 40, and 55 MPa) and temperatures (40, 70, and 95 ºC) on the extraction yield and oil quality of wheat bran oil. Based on the results, the highest antioxidant capacity, alkylresorcinols, and phenolic content of WBO were evaluated at samples obtained using the SFE method at 55 MPa and 95 ºC [71].

Rebolleda et al. used supercritical carbon dioxide at 25.0 ± 0.1 MPa and 40 ± 2 °C to extract wheat bran oil and determine its valuable biologically active compounds. Some of the bioactive compounds are well-known lipophilic or amphiphilic antioxidants, and to obtain WBO extracts enriched with them, all, and to a lesser extent phenolic compounds, were extracted by supercritical carbon dioxide processed as an alternative for the solvent system, to restrict the adverse effects of this method on the composition and the quality of the oily extracts. As reported, WBO showed bioactives including α-linolenic acid, alkylresorcinols, tocopherols, steryl ferulates, and phenolic compounds around 37,47, 7, 18, and 0.025 mg/g oily extract, respectively. As well, the acidity values of the WBO sample were evaluated around 15% oleic acid, while low levels of hexanal and hydroperoxides (around 0.21 ppb and 2.4 meq O_2_/kg, respectively) were found. Chemical analysis of WBO extracts during the darkness condition for 155 days at 21 °C showed a slight decrease in tocopherols and alkylresorcinols contents (20% and13%, respectively) which indicated its stability and potential for industrial applications like nutraceuticals, food ingredients, and others [94].

Jung et al. characterized extracted WBO using hexane and supercritical CO_2_ (SC-CO_2_) at different temperatures (313.15–333.15 K) and pressures (10 to 30 MPa) with a CO_2_ flow rate of 26.81 g/min for 2 h. As reported, peroxide and acid values were lower in SC-CO_2_-extracted oil compared to hexane-extracted oil. Moreover, The oil extracted by SC-CO_2_ showed remarkable radical scavenging activity and antioxidant properties with a higher capability to delay the oxidation by surrounding environment [74].

### Barley

Barley bran is an abundantly inexpensive by-product of barley milling processing and is now mainly used as feed which is rich in starch, protein, minerals, polyphenols, oil, phytosterols, β-glucan, and oryzanol [94, 95]. According to the literature, barley bran can potentially elevate the health-salutary component and antioxidant capacities of foods including crackers, cookies, and bread [96, 97]. Although extraction of protein, β–glucan, and dietary fiber from barley bran has been well studied, there is limited research on barley bran oil, which is an attractive source due to decreasing environmental drawbacks caused by waste disposal and being cost-effective [98].

The physicochemical characteristics of barley bran oil (BBO) were investigated by Zhao et al. According to the results, the barley bran oil extracted with solvent should be further refined before use due to the relatively high peroxide and acid values. Furthermore, the chemical analysis of barley bran oil samples showed 80.12 g/100 g unsaturated fatty acids and 60.41 g/100 g poly-unsaturated fatty acids. Moreover, barley bran oil contained 27 TAGs including LLL, PLL, LLO, and LLLn (21.08 g/100 g, 19.27 g/100 g, 12.24 g/100 g, and 12.17 g/100 g, respectively). BBO also showed 10.74 g/100 g oil, unsaponifiable matter. Additionally, β-sitosterol (5.69 g/100 g oil) was the most predominant phytosterol among other important sterols including campesterol, stigmasterol, and lanosterol (1.32 g/100 g oil, 0.19 g/100 g oil, 0.70 g/100 g oil, respectively) [75].

Qian et al. studied the physicochemical, lipid, and fatty acid characteristics of the bran of hull-less barley from Tibet. Based on the results, the density, refractive index, melting point, acid value, peroxide value, saponification value, iodine value, and un-saponifiable matter of the obtained barley bran oil was evaluated as 0.96 g/cm^3^, 1.41, 30.12 °C, 11.6 mg KOH/g, 19.41 μg/g, 337.62 mg KOH/g, 113.51 mg iodine/g, and 4.5% of total lipids, respectively. The barley bran oil shows a high amount of neutral lipids (94.55% of total lipids), followed by glycolipids and phospholipids (4.20%, and 1.25% of the total lipid, respectively). The oil extracted from barley bran possessed valuable essential fatty acids including linoleic acid followed by palmitic acid (75.08%, and 20.58% of total fatty acids, respectively) as the two major fatty acids in the oil [76].

## Agro-Industrial Waste-Based Essential Oils Applications

Agro-food waste contains hemi- celluloses, starch, pectin, lignin, soluble sugars, protein, cellulose, fat, ash, and flavonoids that are valuable to many yet recovery applications [1, 4]. Essential oils are valuable aromatic compounds that are utilized in flavoring, food industries, cosmetic products (perfumery), and pharmaceutical medicines. In total, thirteen fatty acids were assessed from the plant-based essential oils, using different extraction methods [9]. As concluded, stearic acid as the saturated fatty acid, oleic acid as the monounsaturated fatty acid, and linoleic acid as the polyunsaturated fatty acid are the major acids composition of essential oils, necessary in sufficient amounts for health [4, 9]. Accordingly, the intake of dietary essential fatty acids (linoleic and oleic acids) is promoted by nutritionists due to their significant effect on lowering blood cholesterol levels as well as the etiology of diseases such as cardiovascular disease related to lack of them. Additionally, oleic acid could remarkably increase high-density lipoprotein levels followed by reducing low-density lipoprotein [1, 53].

Based on the literature, the major group of chemicals commonly found in essential oils are ketones (such as menthone, fenchone, carvone, and jasmine), aldehydes (including benzaldehyde, peril aldehyde, and cinnamic aldehyde, representing tranquilizing potentials and anti-septic properties), esters (such as linalyl acetate and lavender, and geranyl formate, possessing strong anti-microbial potentials), terpenes (including limonene, piperine, camphene and pinene, showing strong anti-bacterial, anti-inflammatory, anti-septic, and antiviral activities), hydrocarbons, and alcohols [29]. These compounds could be interesting due to their unique, safe, cost-effective, eco-friendly, biodegradable, and renewable, antifungal, antibacterial, and antiviral properties, making them valuable compounds to be used in different food formulations as flavoring, anti-microbial, and antioxidant properties as well as bioplastics [7]. Anti-microbial food packaging could be developed by incorporating agro-based waste essential oil into different polymer matrixes to reduce, retard, or inhibit the growth of microorganisms for a desired period [3].

## Conclusion

As the demand for nutritional and health-promoting agricultural products increases, the amount of waste produced during industrial processing also elevated. This comprehensive study found an astonishing number of phytochemicals and essential nutrients, including bioactive compounds such as tocopherols, phytosterols, and poly-unsaturated fatty acids, in the post-products of industrial-scale processed agro-foods, including apple, citrus fruits, sour cherry, corn, rice, wheat, and barley processing wastes. Oil scientists continually seek new sources of edible oils to introduce new approaches with remarkable characteristics. Therefore, agricultural-based solid wastes and by-products may be employed to recover essential oils, potentially valuable dietary and cosmetic raw materials. Agro-industrial waste-based essential oils could be obtained by conventional organic solvent method. However, further investigations are needed to consider different extraction methods, such as supercritical fluid extraction and pure CO_2_,… under various conditions as extraction solvents to yield similar active compounds with higher therapeutic effects to maintain optimal health and well-being conditions. Additional analyses using various food models and storage conditions are also suggested to improve the utilization of this EO with functional macro- and micronutrients with antioxidant and antimicrobial properties as a natural alternative for synthetic preservatives.

## Key References

-  Kolanowski W. Perspective of Using Apple Processing Waste for the Production of Edible Oil with Health-Promoting Properties. Appl Sci. 2024;14:2.
  - This article outlines the concept of a technological process for industrial-scale separation of seeds from apple pomace, intended for the production of an edible oil with health-promoting properties
- Lolli V, Viscusi P, Bonzanini F, Conte A, Fuso A, Larocca S, et al. Oil and protein extraction from fruit seed and kernel by-products using a one pot enzymatic-assisted mild extraction. Food Chem X. 2023;19:100819.
  - This article shows the importance of utilizing fruit residues, including seeds/kernels of different fruit varieties, to produce essential oils with high nutritional quality
-  Heydari Koochi Z, Jahromi KG, Kavoosi G, Babaei S. Citrus peel waste essential oil: Chemical composition along with anti-amylase and anti-glucosidase potential. Int J Food Sci Technol. 2022;57:6795–804.
  - This article outlines the antioxidant, anti-glucosidase, and anti-amylase properties of essential oils extracted from different citrus varieties' peels and depicts the impacts of utilizing extracted essential oils in the culinary and medical industries.
- Stryjecka M, Michalak M, Cymerman J, Kiełtyka-Dadasiewicz A. Comparative assessment of phytochemical compounds and antioxidant properties of kernel oil from eight sour cherry (Prunus cerasus L.) cultivars. Molecules. 2022;27:696.
  - The aim of this article was examining the main components and the physicochemical properties of eight cultivars of sour cherry kernel oils. It provides important information about potential possibilities of application of sour cherry kernel oils in pharmaceuticals, offering health benefits.
- Punia S, Kumar M, Siroha AK, Purewal SS. Rice bran oil: Emerging trends in extraction, health benefit, and its industrial application. Rice Sci. 2021;28:217–32.
  - This article discussed various non-conventional techniques for the extraction of rice bran oil, followed by its functionality enhancement by stabilization strategies, and also presented various industrial applications of rice bran oil along with the health benefits in the human body.

## Data Availability

No datasets were generated or analyzed during the current study.
